# Paracetamol and NSAIDs in Cancer Pain Management: Evidence Review and Treatment Considerations

**DOI:** 10.1007/s11864-025-01368-8

**Published:** 2026-02-12

**Authors:** Kate Hanwell, Anna Bradley, Jason W Boland

**Affiliations:** 1 Sheffield Teaching Hospitals NHS Foundation Trust, Herries Road, Sheffield, S5 7AT South Yorkshire United Kingdom; 2 West Middlesex University Hospital, Chelsea and Westminster Hospital NHS Foundation Trust, Twickenham Road, TW7 6AF London, United Kingdom; 3 Wolfson Palliative Care Research Centre, Hull York Medical School, University of Hull, Hull, HU6 7RX United Kingdom

**Keywords:** Paracetamol, Acetaminophen, NSAIDs, Cancer, Pain

## Abstract

Pain is one of the most prevalent and distressing symptoms in cancer, affecting up to 90% of patients and significantly impairing quality of life. Paracetamol (acetaminophen) and non-steroidal anti-inflammatory drugs (NSAIDs) are widely recommended by international guidelines for the management of non-surgical cancer pain, either alone or as adjuvants to opioids. In this paper, the current evidence for their efficacy, tolerability, and safety in this setting is reviewed. Evidence for paracetamol in cancer pain remains limited and of low quality. Small trials and systematic reviews suggest little or no additional analgesic benefit when used alongside strong opioids, and no clear advantage of intravenous over oral paracetamol has been demonstrated. Evidence for NSAIDs is slightly stronger, with studies indicating analgesic benefit both as monotherapy and in combination with opioids, although the quality of evidence is again restricted by small sample sizes, heterogeneity, and outdated trials. Concerns regarding adverse effects, particularly gastrointestinal, renal, and cardiovascular, often limit use, athough short-term use in patients receiving palliative care may be safer than historically perceived. Comparative data between individual NSAIDs, routes of administration, and longer-term use are lacking. Overall, while both paracetamol and NSAIDs are commonly prescribed and theoretically beneficial, high-quality, adequately powered studies in patients with cancer pain are scarce. Further research is needed to evidence their role, especially in opioid-sparing strategies, as well as determining the relative clinical effectiveness and harm of individual NSAIDs in patients with non-surgical cancer pain.

## Key messages

Evidence for the efficacy of paracetamol in cancer pain is weak, with little benefit shown when added to strong opioids.

NSAIDs may provide analgesic benefit alone or with opioids, but the supporting studies are small, dated, and heterogeneous.

Adverse effects of NSAIDs (GI, renal, cardiovascular) remain important, though short-term use in palliative care may be acceptable with appropriate monitoring.

Further high-quality trials are needed to clarify efficacy, opioid-sparing potential, and the role of individual NSAIDs in cancer pain management.

## Introduction

The worldwide incidence of cancer is increasing, with an estimated 20 million new cancer cases and 9.7 million deaths in 2022. This is predicted to rise to over 35 million new cancer cases by 2050 [1]. Pain is a common and distressing symptom in cancer, affecting up to 90% of patients, and being more prevalent in advanced disease [2, 3]. Inadequately controlled pain can negatively impact a patient’s quality of life, causing psychological distress, as well as decline in performance status and possibly even reduced survival [3, 4]. Effective pharmacological management is key, however this is only one facet of cancer pain control. The European Society for Medical Oncology (ESMO) recommends an integrated approach which may also include anticancer treatments, interventional pain techniques and non-invasive therapies such as psychological and rehabilitative interventions [5].

The use of paracetamol and NSAIDs both alone and as adjuncts to opioids in cancer pain is recommended by international guidelines [5–7]. Combining paracetamol and NSAIDs with opioids could have a synergistic analgesic effect due to the different mechanism of actions [8]. This may allow lower opioid doses, ultimately reducing adverse effects [9]. Despite their widely recommended use and theoretical benefit of paracetamol and NSAIDs, evidence for their use in cancer pain is limited [10, 11]. This review assimilates the evidence to provide an up-to-date view on the use of paracetamol and NSAIDs in cancer pain. Literature for this narrative review came from a systematic search of OVID Medline, updated on the 26th September 2025. Terms for cancer, pain, paracetamol and NSAIDs (including the different medications names), were combined and screened by all authors, for inclusion in this review.

## Paracetamol

### Introduction and mechanism of action

Paracetamol is one of the most widely used analgesic drugs worldwide. It is estimated nearly 6,300 tonnes are sold annually in the United Kingdom alone [12]. It is usually well tolerated at therapeutic doses, however it can be hepatotoxic in overdose [13]. Despite this, concerns around its safety in patients with deranged hepatic function have not been substantiated [14]. The mechanism of action of paracetamol is not fully understood [10]. Historically it was believed that the analgesic effects occurred primarily through cyclooxygenase (COX)-dependent inhibition of prostaglandin (PG) synthesis, however recent reviews have suggested that paracetamol has a central mode of action involving the transient receptor potential vanilloid receptor 1 (TRPV1), endocannabinoid, serotonergic, and nitric oxide pathways [15, 16].

### Evidence in cancer pain

Evidence for the use of paracetamol in cancer pain is limited. A 2017 Cochrane review identified three studies with a total of 122 participants. Evidence was rated as very low quality due to small study size and incomplete outcome data. The duration of treatment was less than a week for all studies. All used paracetamol as an adjuvant to strong opioids with median oral morphine equivalent doses of 60 mg, 70 mg, and 225 mg for each of the three studies. It was not clear if paracetamol gave any additional analgesic benefit, which might be due to the high opioid doses [13]. A 2018 meta-analysis of paracetamol in cancer pain (seven studies, with a total of 253 participants) had similar findings. The mean study length was 3.8 days. It concluded that there was no convincing evidence for satisfactory pain relief by paracetamol alone or as an opioid adjunct and found only one study that might potentially favour the use of paracetamol in cancer pain [17]. A 2018 retrospective study of 50 cancer patients who received intravenous (IV) paracetamol demonstrated some effectiveness of paracetamol when used with strong opioids. 38 patients experienced an analgesic benefit from rescue administration of IV paracetamol, measured by an improvement in pain scores on a 4-point verbal rating scale. The background opioid dose used for pain control was found to be associated with the efficacy of the rescue administration of IV paracetamol, with IV paracetamol improving pain in patients taking under 45 mg/day of oral morphine equivalent [18]. The results of this study should be viewed speculatively, given small sample size and the uncontrolled retrospective study design. A subsequent 2023 randomised controlled trial (RCT) evaluated the role of paracetamol in hospitalised cancer patients with moderate to severe pain who were receiving strong opioids. 112 patients were recruited and randomised to either placebo or IV paracetamol. The primary outcome measure of the study was pain intensity difference between baseline and 48 h. Secondary outcomes looked at change in morphine equivalent daily dose and patient perception of pain control. At 48 h there was no difference between groups in pain intensity, morphine equivalent daily dose or patient perception [19].

### Route of Administration

There is debate about increased efficacy of IV paracetamol vs. oral, due to potential pharmacokinetic benefits. There are no studies comparing different administration routes in non-surgical patients with cancer. Studies from other settings, such as the emergency department (ED) and postoperatively give some insight into effectiveness. A 2015 systematic review of 6 small RCTs found no differences in efficacy between the two preparations. The pharmacokinetics of IV paracetamol were predictable and reliable, with higher bioavailability, more rapid increase in concentrations and a higher T_max_ than oral paracetamol. However, there was no evidence that these made IV paracetamol a more effective analgesic than oral paracetamol [20]. A 2017 RCT compared IV and oral paracetamol as an opioid adjunct in the management of moderate to severe pain in the ED setting (*n* = 87). There was a small but clinically significant improvement in pain in both groups although there was no difference between IV and oral preparations [21]. A 2021 scoping review of IV paracetamol in the postoperative setting, reported conflicting results with no consistent clinical benefit when compared to other paracetamol preparations [22]. A 2021 retrospective analysis of 353 consecutive gynaecological oncology surgery patients reported no difference in postoperative (days 0 or 1) opioid consumption between preoperative IV and oral paracetamol [23].

## Non-Steroidal Anti-inflammatory Drugs (NSAIDS)

### Introduction and Mechanism of Action

NSAIDs are widely prescribed in many medical conditions with the aim of providing anti-inflammatory, antipyretic and analgesic effects in both acute and chronic pain [24]. Prevalence of NSAID use in cancer pain varies from 4 to 19% [25]. In determining the superiority of one NSAID over another in managing cancer pain, the evidence on the efficacy of NSAIDs and any potential harms must be evaluated. NSAIDs exert their action through their inhibition of the cyclooxygenase (COX) enzymes preventing the synthesis of prostaglandins, prostacyclins and thromboxanes [24]. There are two COX isoforms, COX-1 and COX-2. COX-1 is constitutively expressed and responsible for numerous physiological functions [24]. In contrast, COX-2 expression is much more tightly regulated and can be induced by cytokines and other inflammatory mediators in several tissues [24].

**Fig. 1:**
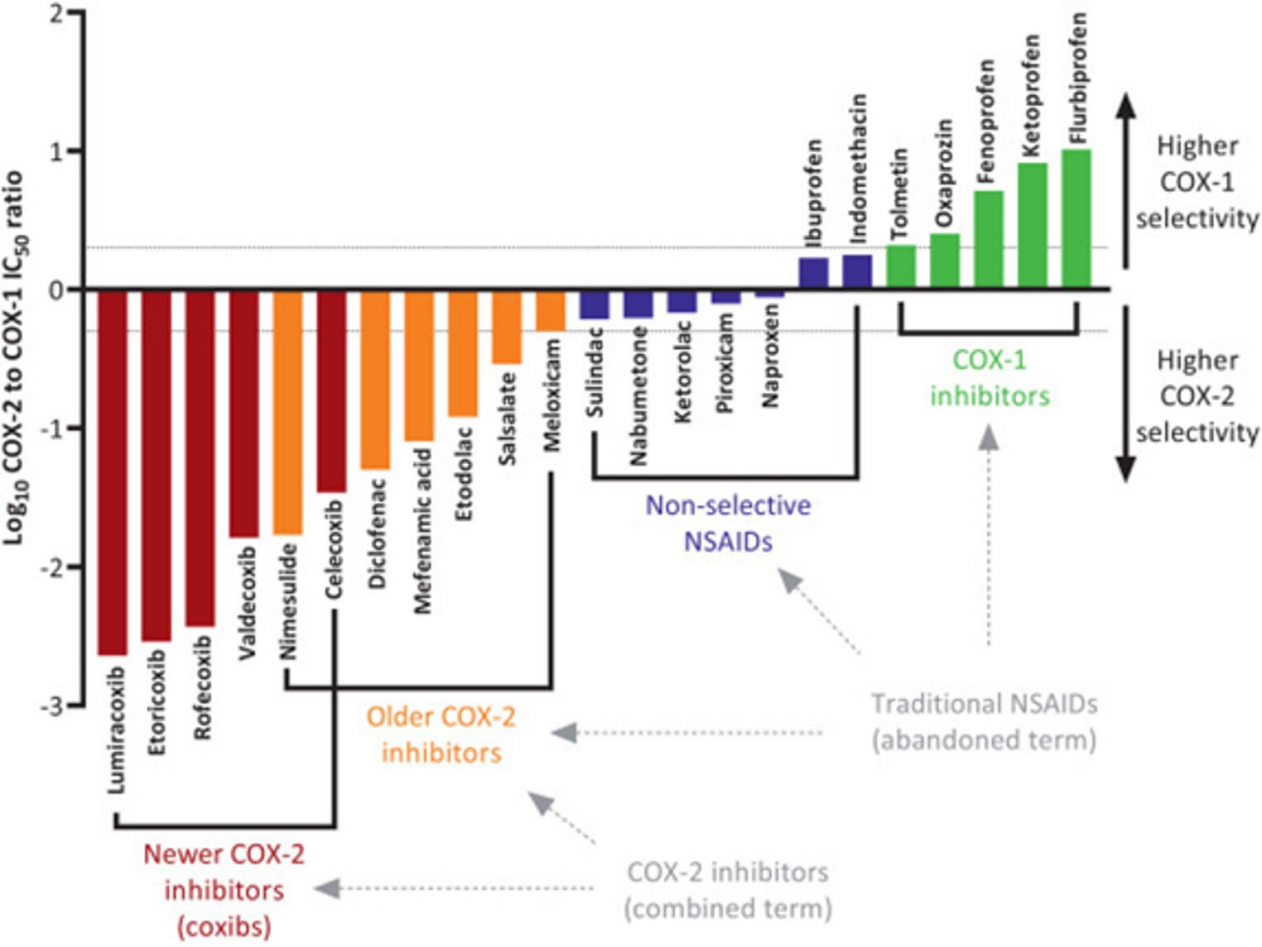
Range of COX selectivity for NSAIDs. Reproduced with permission from *Recategorization of Non-Aspirin Nonsteroidal Anti-inflammatory Drugs According to Clinical Relevance: Abandoning the Traditional NSAID Terminology*, Canadian Journal of Cardiology 2021; 37 [11]: 1705–1707 [26]. License: https://creativecommons.org/licenses/by/4.0/

Figure 1 shows the varying COX selectivity between commonly used NSAIDs. It had been thought that COX-2 specific inhibitors would provide anti-inflammatory and analgesic effects with a reduced side effect profile, however two COX-2 inhibitors, rofecoxib and valdecoxib, were withdrawn in the early 2000s due to increased risk of cardiovascular and cerebrovascular events [25]. Other COX-2 inhibitors remain available, but all NSAIDs are associated with a degree of GI and cardiovascular risk. Therefore, when prescribing an NSAID clinicians must weigh these risks against the potential benefits of managing cancer pain. This is discussed further below.

### Evidence in Cancer Pain

When considering the role of NSAIDs in cancer pain, it is important to consider their role as a single analgesic or in combination with opioid therapy.

#### Evidence for NSAIDs as Monotherapy

A meta-analysis in 2018 identified 26 studies with 2252 participants. The studies examined various NSAIDs, including diclofenac, naproxen, ketorolac, piroxicam, ketoprofen and the COX-2 inhibitors celecoxib and nimesulide. They concluded that there was substantial evidence regarding the efficacy of NSAIDs compared with placebo for cancer pain, and overall quality of evidence on withdrawals due to inadequate pain relief, and adverse events ranged from moderate to high. The doses, routes of administration and duration varied throughout the analysed studies [17]. A 2019 systematic review included seven studies with a total of 509 participants that examined the effects of NSAIDs versus placebo. Again, they found they all demonstrated the benefit of NSAIDs over placebo, with adverse effects being comparable between groups. However, all studies were single dose and were published before 1991 [25].

#### Evidence in Combination with Opioids

The results from a 2017 Cochrane review, using mean data for the most part, indicate that NSAIDs in combination with opioids reduce pain to an acceptable level (≤ mild) in a proportion of participants, but the large standard deviations show that the response is variable. The treatment duration in all studies lasted at least one week. However, most studies had a high risk of bias due to issues with blinding, incomplete outcome data, or small sample sizes, resulting in very low-quality evidence [27].

A meta-analysis in 2018 identified 6 studies that compared NSAIDs with opioids, three with morphine and three with weak opioids, however the strength of the morphine studies was questioned due to small numbers of participants and high dropout rates. No significant difference between NSAIDs and opioids was seen between 2 of the 3 studies looking at weak opioids. Six studies were identified with NSAIDs used as a co-analgesic to strong opioids but these again only had small sample sizes (142 participants across 6 studies) [17].

A 2019 systematic review identified 5 studies that looked at NSAIDs alone versus in combination with an opioid. It is worth noting that none of these studies included COX-2 inhibitors, none were conducted within the past 20 years and only 2 studies used more than a single dose of NSAID. Only one study reported a statistically significant finding that the combination of NSAID and opioid provided superior analgesic effect. Three studies demonstrated some opioid sparing effect of NSAIDs; however they were too small and underpowered to make firm recommendations [25].

A 2022 systematic review looked at the opioid sparing effects of ketorolac in palliative care patients receiving opioids for chronic cancer related pain. 9 studies were included, although significant heterogeneity was noted. Only one RCT showed significant reductions in the observed total daily dose of morphine, but the strength of evidence was low [28].

#### Evidence Comparing Different NSAIDs

This review found that the evidence comparing different NSAIDs was very limited. A 2017 Cochrane review attempted to do this but found no comparative analyses were possible [27]. The meta-analysis in 2018 weighed all NSAIDs as a group and not individually [17]. A systematic review in 2019 found only 2 studies, both with small sample sizes, published in the last 20 years. There was no significant difference demonstrated between NSAIDs [25].

### Route of Administration

NSAIDs come in different forms and can be delivered by various routes, including IV, sustained-release oral preparations, topical gels, patches, and suppositories [29]. This review found no studies that have compared route of administration of NSAIDs on their efficacy and adverse effects directly in adults with cancer pain. There is limited data in cancer pain, and limited recent data, so other potentially relevant (albeit old) reviews have been included.

A 1998 review assessed the evidence for a difference in analgesic efficacy and adverse effects of NSAIDs given by different routes, primarily rectal, oral, IV or IM, in several chronic and acute pain conditions [30]. It concluded in all other pain conditions, apart from renal colic where IV administration was superior, there is a lack of evidence of any difference between routes. Oral NSAIDs should be used when patients can swallow. Although the age of this study may limit its applicability. more recent reviews evaluating the use of NSAIDS and comparing routes of administration in settings such as acute postoperative pain [31] and renal colic in the ED setting [32] continue to support this view.

The topical versus oral route has primarily been evaluated in musculoskeletal (MSK) pain. Topical NSAIDs aim to reduce systemic exposure [29]. A Cochrane review published in 2015 found some formulations of topical diclofenac and ketoprofen were beneficial in acute pain conditions such as sprains or strains, with low Number Need to Treat (NNT), values between 1.8 and 4, and probably provided similar pain relief to that provided by oral NSAIDs [33]. However, a further 2016 Cochrane review found that in chronic MSK conditions with assessments over 6 to 12 weeks, topical diclofenac and ketoprofen had limited efficacy in hand and knee osteoarthritis [34].

Certain NSAIDs such as ketorolac and parecoxib can be administered subcutaneously; studies evaluating this route are limited. A 2015 review evaluated the safety and efficacy of subcutaneous (SC) administration of ketorolac for pain management. It analysed case reports and observational studies in 91 patients with cancer pain. In addition, it analysed 2 RCTs in the setting of surgical procedures. It concluded SC administration of ketorolac appeared to provide analgesic benefit in both cancer-related and postoperative pain. It was shown to have minimal adverse effects regarding GI or renal issues and appeared to be a safe option when no other route of administration is available [35].

Parecoxib (a selective COX-2 inhibitor) can also be administered SC. A retrospective chart review of 80 palliative care patients with cancer pain receiving parecoxib via continuous sub-cutaneous infusion (CSCI) found a statistically significant difference when looking at the reduction in pain scores and number of PRN opioid doses used. It found that parecoxib was generally well tolerated and the overall conclusion was that parecoxib is useful in cancer pain when oral administration is no longer possible [36].

### Side Effects of NSAIDs in Patients with Cancer Pain

There is limited data on the adverse effects of NSAIDs specific to patients with cancer pain, however side effects of chronic NSAID use are well documented in non-malignant, mainly rheumatological, conditions [37]. A 2013 meta-analysis highlighted the major side effects and the variation between different NSAIDs with an emphasis on the gastrointestinal and vascular side effects. Death from vascular events was increased significantly by COX-2 inhibitors and diclofenac (an additional 3 per 1000 deaths per year compared to placebo), non-significantly by ibuprofen and not by naproxen [38]. All NSAIDs doubled the risk of heart failure leading to hospital admission. NSAIDs increased the risk of upper gastrointestinal complications by around 2–4 times; however, COX-2 inhibitors yielded the lowest risk of such complications [38]. This review included a range of COX-2 inhibitors, including the discontinued rofecoxib. Some strategies are commonly adopted in an attempt mitigate some of the risk, for example prescribing with a proton pump inhibitor or adjusting the timing when taking aspirin alongside an NSAID to reduce risk of gastrointestinal complications [29].

The risk of renal harm from NSAIDs is well known [39]. A 2023 international prospective consecutive cohort pharmacovigilance study (92 patients, 91% had cancer), suggested that the risk of acute kidney injury (AKI) with NSAIDs is more associated with hypovolaemia and concomitant medications than baseline renal function alone. It suggested that NSAIDs are often used in patients with significant risk factors for bleeding and AKI, but that a significant proportion of that risk comes from drug-drug interactions and is therefore potentially avoidable [40]. In this study, 83% of patients reported benefits from NSAIDs at 14 days. 22% reported adverse effects, including nausea (8%), vomiting (3%), acute kidney injury (3%), and non-gastrointestinal bleeding (3%), but importantly no patients discontinued their NSAIDs due to adverse effects [40]. Not all harms were thought to be due to NSAIDs with one patient developing an AKI due to cancer related obstructive uropathy. In practice, risks can often be mitigated with a proton pump inhibitor, short-term use, and patient selection. When considering whether to prescribe NSAIDs in cancer pain, the prescriber must weigh up potential benefits against risks for the individual patient.

## Future Studies

There is a significant paucity in recent review articles and RCTs for both paracetamol and NSAIDs in non-surgical cancer pain management. New and high quality RCTs are therefore needed to clarify their effectiveness. An RCT to evaluate stopping paracetamol in patients also on strong opioids for moderate to severe cancer pain is underway [41]. For NSAIDs, in a survey of palliative medicine physicians, an RCT of NSAIDs as opioid adjuncts for cancer related bone pain was suggested to be the most pragmatic design for future studies [37].

## Conclusion

Cancer pain remains a major clinical challenge, and despite widespread use of paracetamol and NSAIDs, the evidence base to support their effectiveness is limited (Table 1). For paracetamol, available trials are few, underpowered, and largely inconclusive, suggesting minimal additional benefit when combined with strong opioids. NSAIDs appear to have greater potential utility, with some evidence of analgesic benefit both alone and as adjuvants. However, the studies are heterogeneous, often dated, and insufficient to guide firm recommendations regarding specific drugs, optimal dosing, or duration of treatment.

**Table 1:** Summary of evidence

Drug	Efficacy	Safety/tolerability	Strength of Evidence	Overall conclusion
Paracetamol	No clear evidence of meaningful benefit for cancer pain, either alone or in combination with opioids. No benefit of intravenous over oral in other patient groups	Generally well tolerated and safe; hepatotoxicity risk with overdose	Low. Low quality small, old and inconsistent trials with poor study design, heterogeneity and underpowered	Current evidence does not support routine use; role remains uncertain
NSAIDs (e.g., ibuprofen, diclofenac, celecoxib)	Some evidence of analgesic benefit, especially in bone/inflammatory pain. Possible opioid-sparing effect	Short-term use may be safer than historically perceived; risks include gastrointestinal, renal, and cardiovascular adverse events, particularly in frail patients. Mitigated by short-term use and proton pump inhibitors for gastrointestinal toxicity	Low/moderate. Small, heterogeneous old trials. Lack of large contemporary RCTs; limited head-to-head comparisons	NSAIDs may have a role, especially short-term, but require careful patient selection and monitoring

Safety remains a concern, particularly with NSAIDs, given risks of gastrointestinal, renal, and cardiovascular toxicity. Nonetheless, short-term use in patients receiving palliative care may be more acceptable than in chronic non-malignant pain, provided patients are carefully selected and monitored. Current practice relies on clinical judgement, balancing potential benefits with individual risk factors and preferences.

Future research should prioritise high-quality randomised controlled trials that assess clinically meaningful outcomes, such as pain relief, opioid-sparing effects, quality of life, and tolerability. Comparative studies between NSAIDs, routes of administration, and defined patient subgroups are especially needed. Until then, clinicians must use paracetamol and NSAIDs pragmatically, as part of a multimodal and individualised approach to cancer pain management.

## Key References

- Leiva-Vásquez O, et al. Is Acetaminophen Beneficial in Patients With Cancer Pain Who are on Strong Opioids? A Randomized Controlled Trial. J Pain Symptom Manage. 2023 Sep 1;66(3):183–192.e1.
  - ◦This placebo-controlled randomized controlled trial specifically investigates the added benefit of intravenous (IV) paracetamol in cancer patients already receiving strong opioids. It showed no difference between groups, challenging the routine use of paracetamol in this population and informing evidence-based decisions on adjunctive analgesic therapy.
- McNeill R, et al. Non-steroidal anti-inflammatory drugs for pain in hospice/palliative care: an international pharmacovigilance study. BMJ Support Palliat Care. 2023 Dec;13(e3):e1249–57.
  - ◦An international pharmacovigilance study analysing NSAID use in hospice and palliative care settings, providing real-world safety data critical for clinicians managing cancer pain in end-of-life scenarios. This research highlights the low incident of, and patterns of NSAID-related adverse events, informing safer pain management strategies in palliative care.
- Torpen LN. PARASTOP - Paracetamol with Strong Opioids in Moderate to Severe Cancer Pain. 2022. https://www.hra.nhs.uk/planning-and-improving-research/application-summaries/research-summaries/parastop-paracetamol-with-strong-opioids-in-moderate-to-severe-cancer-pain/.
  - ◦The PARASTOP is a clinical trial investigating the efficacy and safety of combining paracetamol with strong opioids for moderate to severe cancer pain, by randomising people to either stop using paracetamol (placebo) or continue paracetamol. This ongoing research offers promising insights into the role of paracetamol in patients with cancer pain taking strong opioids.

## Data Availability

No datasets were generated or analysed during the current study.
